# A randomized trial to reduce sugar-sweetened beverage and juice intake in preschool-aged children: description of the Smart Moms intervention trial

**DOI:** 10.1186/s12889-016-3533-8

**Published:** 2016-08-19

**Authors:** Brooke T. Nezami, Leslie A. Lytle, Deborah F. Tate

**Affiliations:** 1 Lineberger Comprehensive Cancer Center, University of North Carolina at Chapel Hill, Campus Box #7294, Chapel Hill, NC 27599-7294 USA; 2 Department of Health Behavior and Nutrition, Gillings School of Global Public Health, University of North Carolina at Chapel Hill, Campus Box #7440, Chapel Hill, NC 27599 USA

**Keywords:** Early childhood, Obesity, Sugar-sweetened beverages, Maternal obesity, Intervention

## Abstract

**Background:**

Obesity in young children remains a public health concern, and maternal weight is one of the strongest predictors of obesity in early childhood. However, parental adherence in interventions for young children is often low and existing programs have had mixed success. An innovative approach to treatment is needed that increases adherence among mothers and improves weight-related behaviors simultaneously in mothers and children. The objective of the Smart Moms randomized controlled trial (RCT) is to test the efficacy of a 6-month primarily smartphone-delivered program to reduce sugar-sweetened beverage and juice consumption among children ages 3–5 whose mothers are overweight or obese. This paper describes the study design and intervention.

**Methods/Design:**

Mother-child dyads were eligible if the mother was overweight or obese, owned a smartphone, and if the child was between the ages of 3–5 and consumed 12 oz or more per day of sugar-sweetened beverages (SSBs) and 100 % fruit juice. Participants were randomly assigned to the Smart Moms intervention or a waitlist control group. The intervention consisted of theoretically grounded and evidence-based behavioral strategies delivered through one group session, lessons on a mobile-optimized website, and text messages. Mothers submitted self-monitoring information via text message and received regular tailored feedback emails from interventionists. The primary outcome is change in child SSB and juice consumption and a secondary outcome is change in maternal weight.

**Discussion:**

This Smart Moms study was designed to determine if a low-burden intervention delivered using mobile methods and targeted towards mothers could be effective at changing child sugar-sweetened beverage intake. Results will indicate if mobile-based methods can be a feasible way to engage mothers in family-based studies and will inform successful strategies to prevent childhood obesity through parent-targeted approaches.

**Trial registration:**

Clinicaltrials.gov NCT02098902 (Registered March 25, 2014).

## Background

Over the last 40 years, research has confirmed a trend for the onset of obesity at increasingly younger ages. One in three low-income children are overweight by age 5 [1] and the majority of children who become overweight or obese reach overweight status before the age of 5 [2]. This is concerning because excessive weight gain in early childhood is known to increase the risk of obesity and its health-related consequences throughout childhood and into adulthood. To reduce the risk of obesity, it is imperative to focus on increasing children’s healthy weight behaviors and preventing weight gain beginning at an early age.

### Maternal weight and sugar-sweetened beverage consumption as risk factors

While there are many determinants of early childhood obesity, some of the strongest and most consistent predictors are maternal weight and weight-related behaviors [3, 4]. Thus, children of overweight and obese mothers are at particularly high risk for obesity during their childhood years. Before the age of five, children are socialized primarily in the family environment with parents serving as “gatekeepers” in the home by providing specific diet and activity opportunities for children. Intervening with overweight and obese mothers to make the home environment healthier and make dietary changes for themselves and their children could reduce the risk of the child becoming overweight.

The child’s own dietary behaviors, above and beyond those associated with maternal weight and diet, are also predictive of child overweight [5]. Sugar-sweetened beverage (SSB) intake is one of the dietary factors most consistently associated with early childhood obesity across studies [4, 6]. NHANES data indicates that, on average, children in this age range are consuming an average of 70 cal a day from sugary drinks including caloric carbonated beverages, fruit drinks, and sport drinks [7]. This increases to 176 calories a day when including consumption of 100 % fruit juice [8]. Intervention evidence in children ages 4–12 has shown that replacing SSBs with noncaloric beverages can lead to a lower increase in BMI z-scores over 18 months compared to children making no reduction in SSBs [9]. Thus, intervening with mothers to reduce child sugar-sweetened beverage consumption seems to be a promising target for intervention to prevent early childhood obesity.

### Existing parent-targeted interventions

Interventions among school-aged children and among preschool-aged children within childcare centers and preschools have shown that the active involvement of the parent is a critical factor for success [10–12]. But unfortunately, interventions requiring parent involvement often have low adherence and high dropout rates. For example, in a study that targeted mothers and their preschool-aged children that did not result in changes in child diet, physical activity, or sedentary behaviors, participants participated in half of the required phone calls, less than half of the participants attended the group session, and 68 % completed the follow-up assessment at 12 months [13]. While there have been several parent-targeted interventions that have successfully led to changes in young children’s diet or physical activity behaviors, participation in these interventions has also been low and successful behavior change among parent–child dyads was associated with greater treatment adherence [13–16].

Even in studies that have targeted mothers only with no focus on changing the child’s behavior, attendance and retention are typically low, particularly among those that have required face-to-face group meetings [17]. As an example, a study with mothers of children ages 1–4 that involved 8 weekly 2-h classes had only 44 % of participants in attendance at the first session, and attendance continued to decrease over time [18]. In a large RCT of postpartum women with 18 in-person group meetings and additional telephone calls focusing on healthy eating and physical activity, the group meetings had only 27 % attendance. Overall, attendance at group meetings was positively associated with weight loss [19].

### Intervening with mothers of young children to reduce child SSBS and juice

Young children of overweight and obese mothers often engage in behaviors similar to their mothers, including SSB consumption, and thus are at high risk of becoming overweight or obese [20, 21]. Moreover, mothers of young children are more likely to be overweight or obese due to the time demands and life transitions that occur when having children [22, 23]. Targeting mothers who are overweight or obese as the agent of change in the household could be a promising way to improve child dietary behaviors while also helping the mother lose weight. Because mothers face many barriers to weight control behavior change, including time demands and lack of childcare, there is a need for interventions that are uniquely targeted to the needs of mothers and children. A smartphone-based intervention that requires relatively few in-person visits might increase adherence by reducing mothers’ travel time, providing a flexible schedule, and minimizing the amount of time spent participating in study activities.

Interventions using text messages and mobile-based websites have the capability to reach many people, as mobile phone use is high and continues to increase among adults in the United States. Among the 91 % of Americans who own a cell phone, 63 % of them use it to access the internet or email [24]. There has been a widespread adoption of eHealth and mHealth interventions in recent years, many of which have been effective in producing clinically meaningful changes in behaviors and weight loss [25–28]. However, surprisingly few studies have used the internet and/or text messages to reach parents of young children, and there have been no mobile-based studies that have targeted a parent to reduce their child’s SSB/juice intake. A critical next step in the literature is to use innovative treatment approaches that might increase adherence among mothers in order to improve weight-related behaviors in both mothers and children. Thus, the objective of this study is to test the efficacy of a 6-month smartphone-based program that targets mothers who are overweight or obese to reduce the SSB and juice consumption of their preschool-aged children compared to a waitlist control group.

## Methods/Design

### Study design

Smart Moms is a two-group randomized controlled trial designed to test the efficacy of a 6-month program to reduce SSB and juice consumption among children ages 3–5 of mothers who are overweight or obese compared to a waitlist control group. Mother-child dyads were randomly assigned to the Smart Moms intervention group or to a waitlist control group after baseline data were collected. Randomization was stratified by two factors: whether or not the child was in childcare/kindergarten and child baseline SSB/juice intake. The primary hypothesis is that the Smart Moms program will result in a greater reduction in child fluid ounces of SSBs and juice compared to the control group at 6 months. Secondary outcomes include maternal percent weight loss, maternal caloric beverage consumption, and child BMI z-score at 6 months. The study was approved by the UNC non-biomedical IRB on March 18, 2014.

### Eligibility criteria

The eligibility criteria were designed to target a population of children with a high risk of obesity, particularly those who have a high consumption of SSBs and juice (SSB/juice) and have mothers who are overweight or obese. The eligibility criteria included maternal BMI of 25–50 kg/m^2^ and a child between the ages of 3–5 who consumed at least 12 oz a day of SSBs/juice (including 100 % fruit juice, fruit drinks, sport drinks, caloric sodas, sweetened teas, and flavored milks). Mothers were required to have a smartphone with an active data and text-messaging plan. Exclusion criteria for mothers included: participation in another weight loss program, pregnancy within the last 6 months, planning to become pregnant in the next 6 months, planning on moving from the study area within the 6-month study period, maternal diagnosis of schizophrenia, bipolar disorder or hospitalization for a psychiatric diagnosis in the past year, or unable to participate in alternatives to sedentary behavior including standing and walking. Children were not eligible if they had a medical condition that would preclude changes in dietary intake or physical activity.

### Recruitment

**Fig. 1:**
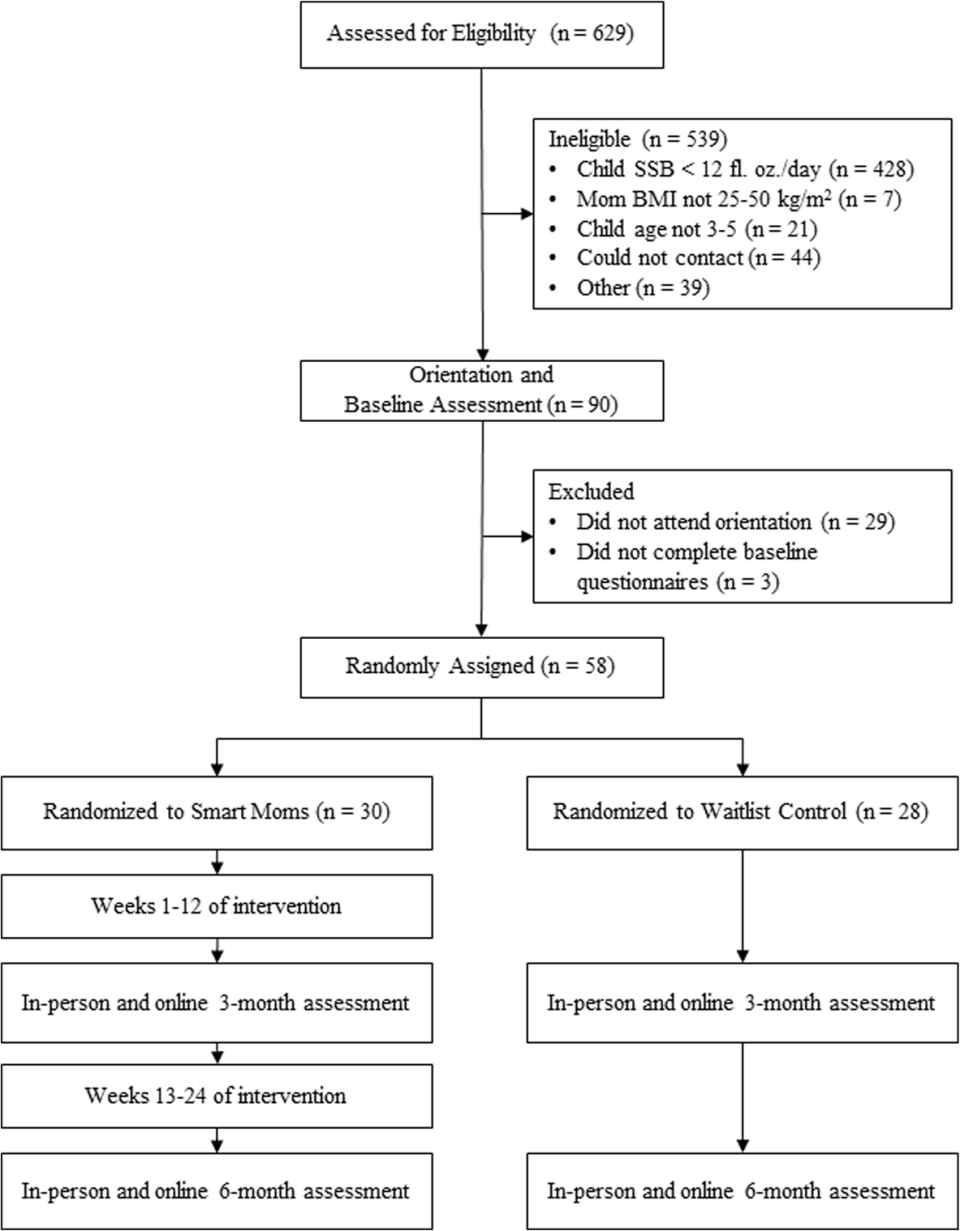
Flow of participants

Participants were recruited from several counties within and surrounding a large metropolitan area in North Carolina, USA. Recruitment methods included flyers, contact with daycares, pediatricians’ office, and WIC clinics, in addition to online advertising on local parents’ websites and Facebook. Interested participants completed an online screening survey to determine their eligibility. Women who met initial eligibility requirements were then followed up by phone call for additional screening. Fully eligible participants attended an in-person orientation session at a university-based research clinic where they provided written informed consent to be in the study to the principal investigator and completed the baseline assessment. Figure 1 depicts the flow of participants from recruitment through the end of the study.

### Sample size and statistical analysis

The primary outcome for the study was change in child SSB/juice intake in fluid ounces from baseline to 6 months. Based on evidence from three previous studies that resulted in significant SSB/juice reductions in children [14, 29, 30], the study was powered to detect a difference of a 6-oz reduction between groups at 6 months, with a within-group standard deviation of 6.0. Therefore, with 80 % power at an alpha level of .05 and accounting for 20 % attrition, the study required 21 participants per group for a minimum of 42 total participants to detect a significant difference in SSB/juice reduction.

### Intervention

The Smart Moms intervention was developed based on Social Cognitive Theory (SCT), which proposes that individual, social and environmental factors interact in a reciprocal manner (i.e. reciprocal determinism) to explain behavior [31]. The Smart Moms intervention included behavior change strategies that specifically targeted the following constructs from SCT that have been associated with sugar-sweetened beverage and juice consumption in previous studies: self-efficacy, outcome expectations, perceived barriers, self-regulation, observational learning, and aspects of the home environment (Table 1). In addition, the intervention targeted positive reinforcement and limit setting within the home, as they are additional strategies that are associated with family behavior change [32]. The overall goal was to use SCT to target the behavior of mothers of young children to, in turn, promote behavior change in the child.

**Table 1:** Mapping theoretical constructs to behavioral intervention strategies

Determinant	Strategies
Self-efficacy	Prompt self-monitoring Set short-term goals Provide tailored performance feedback
Outcome Expectations	Provide general information about risk of obesity in young children Provide general information on relationship between SSBs and obesity
Perceived Barriers	Prompt barrier identification Provide strategies to overcome barriers Prompt behavior change with cues to action/encouragement
Self-Regulation	Prompt self-monitoring Set short-term goals
Home Environment	Environmental restructuring
Limit Setting	Provide tailored performance feedback Stress management
Positive Reinforcement	Provide contingent rewards Praise child for behavior
Observational Learning	Provide information on mother as behavioral role model for child

#### Weekly goals

The long-term beverage goal for children was to consume no more than 4 fluid ounces of SSB/juice per day. An additional recommendation was to limit the 4 oz per day to 100 % fruit juice while eliminating all other sugar-sweetened beverages (i.e. fruit drinks, sweetened tea, flavored milk, regular soda, sports and energy drinks). The child’s goal for the first week was similar to their baseline level, but was gradually reduced to 4 oz per day (4 fl. oz. of SSB/juice = 1 serving for children) by week 8 of the program and remained there for the rest of the program (e.g. setting graded tasks). Mothers’ weekly beverage goal for the duration of the program was 8 oz or less (8 fl. oz. caloric beverages = 1 serving for mothers) of caloric beverages per day, which included all sweetened beverages described above plus milk (plain and sweetened), coffee with any caloric additions, and alcoholic drinks. An 8-oz. liquid measuring cup was provided to mothers so that they could measure exact 4-oz. (for children) or 8-oz. (for adults) beverage servings for the first week of the program in order to become accustomed to serving sizes. Mothers were also asked to limit the number of “red zone” foods they ate per day, which are high-calorie, high-fat foods from the Traffic Light Diet [33]. They were only asked to limit how many *times* per day they ate a red food, but were not asked to calculate calories or serving sizes. Their weekly red zone foods goal was set at half of their baseline intake. Mothers who were not consuming many caloric beverages at baseline, and thus did not have large beverage changes to make, were asked to put a greater emphasis on reducing their red zone foods. Because detailed measuring and self-monitoring of calories is time consuming, the simplified manner of measuring food and beverage intake in this study was intended to promote adherence to positive dietary changes and continued self-monitoring, as described below. Lastly, mothers had a goal to weigh themselves every day. Scales were not provided to participants, but participants who did not already have a scale were encouraged to purchase one.

#### Self-monitoring

Throughout the intervention mothers self-monitored their weight, their caloric beverage consumption, their intake of red zone foods, and their child’s SSB/juice consumption. Rather than tracking all calories, mothers checked a box in a simple, study-provided paper log each time they had a serving of a caloric beverage, each time they had a red zone food (regardless of caloric content), and each time their child had a serving of SSB/juice. At the end of each week, participants received a text message with a prompt to submit their weekly totals of caloric beverages servings, red zone foods, their last reported weight, and their child’s weekly total of SSB/juice.

#### Kick-off group session

Mothers randomized to the intervention condition attended a 75-min in-person kick-off session at the beginning of the study. They received information about the weight-related and dental consequences of consuming too many sugar-sweetened and non-nutritive beverages for both children and adults. Mothers were given their weekly goals and their child’s weekly goals and instructed on how to track their behaviors using the diaries and text messages. The session emphasized to mothers that making small, simple changes in their beverages and red zone foods would result in a reduction of 100–300 cal per day, which could lead to modest weight losses after several months. Mothers were encouraged to view their own weight loss efforts as a way to make healthy behavior changes in the home environment and be a positive role model for the benefit of their children’s health.

Participants were sent home with a “toolkit” of study materials, which included a printed copy of the first lesson, a chart with the mother’s and child’s personalized weekly beverage goals (gradual tailored progression), weekly self-monitoring diaries, sticker charts, stickers, and weekly prizes (described below), two 6-oz. cups with lids simple measuring of their child’s beverages, water bottles for the mothers and children, an 8-oz. liquid measuring cup, and a User’s Guide with program timelines and instructions on how to use the mobile website and text messaging components of the program.

#### Website and lessons

The Smart Moms website was created to provide short lessons and resources to help mothers change their own dietary behaviors and to work with their children to reduce their SSB/juice consumption. The website was responsive, or mobile-optimized, with content that was easily viewable on a smartphone so that mothers could access the information at any time. There were a total of 18 lessons that were updated weekly for the first 12 weeks and biweekly for weeks 13–24. They included information on behavioral strategies to reduce caloric beverages and red foods, but also incorporated topics on dietary intake, reading food labels, snacking, sleep, physical activity, screen time, communication and problem solving with their children, stress management, and relapse prevention strategies. Participants received a text message with a link to the study website indicating when the newest lesson had been posted.

#### Weekly tailored feedback

The weekly self-monitoring data submitted through text message were used to send participants tailored feedback. They received this personalized feedback through email, weekly for the first 12 weeks and biweekly for weeks 13–24. Generally, feedback messages were tailored to whether or not the child met their beverage goal for the week, whether or not the mother met her beverage goal for the week, whether she met her red foods goal for the week, and mothers’ weight loss progress.

#### Monthly progress check-ins

At the end of each month, participants received a link to a brief questionnaire that asked them to report which of the lessons they had read in the last month. Additionally, for goals they were not regularly meeting, they were asked to report the biggest barrier to change they experienced in the last month. This specific barrier was used to provide additional tailored feedback with strategies on overcoming the barrier in the next feedback email.

#### Tips, motivational messages, and goal progress assessment text messages

In addition to the lesson and self-monitoring prompts, all participants received an additional two text messages a week during the first half of the program and one additional text message per week during the second half of the program. These consisted of tips for behavior change, motivational messages, and goal progress assessments, which were brief multiple-choice questions about the progress that mothers were making towards one of the behaviors that week. Based on their answer, a tailored feedback message was sent in response that usually consisted of encouraging them to continue their good progress or providing them with support if they were having difficulty. These messages were sent at random intervals, as that has been shown to increase the effectiveness of SMS-based interventions [34].

#### Child reinforcement charts

Mother/child dyads were given 12 colorful, engaging weekly charts to track the child’s beverages together with their child as well as stickers and 12 token prizes (e.g. $1 toys, games, etc.). Each chart stated the child’s beverage goal for that week, and the mothers were instructed to have their child put a sticker on each day that they met their beverage goal. If the child met their weekly beverage goal, the mother gave them a prize at the end of the week. Although there was no specific goal for water consumption, the study also provided mother-child dyads with additional sticker charts to encourage the child to drink more water. Children placed a sticker on the chart each time they had a glass of water and mothers were encouraged to give them a prize when the chart was full. The charts and prizes served to increase positive reinforcement for the child’s SSB/juice and water changes during the first half of the program. The study did not provide these charts and prizes after week 12, but mothers were encouraged to use the same principles and to continue with charts and prizes of their own throughout the remainder of the program.

### Control group procedures

The modified waitlist control group attended one group session where they received their randomization assignment and were encouraged to continue with their normal behaviors for the next 6 months. Because of the nature of a control group, contact was limited with control group participants. However, they completed assessment visits at the same time as treatment group participants and received incentives (described below) for doing so. The face-to-face contact they received with the principal investigator at the 3-month assessment may have encouraged retention at 6 months. They received a modified version of the intervention following the 6-month assessments.

### Measures

Participants completed assessments at baseline (prior to randomization), 3 months, and 6 months. Each assessment period included an in-person visit and the completion of online questionnaires. Participants in either treatment group who missed assessment visits were contacted frequently by phone and email to schedule and complete assessment visits. Participants received a $20 honorarium for completing each of the 3- and 6-month assessments, meaning participants who completed both assessments received $40 total.

#### Demographics

A lifestyle questionnaire assessed demographics at baseline, including age of the mother and child, race/ethnicity, income, maternal employment status, maternal education, marital status, number of children in the home, and whether the child was in childcare or kindergarten (hereafter referred to as childcare status).

#### Anthropometrics

Trained research staff measured the height and weight of both mothers and children in light clothing (without shoes). Due to limited staffing, the principal investigator, who was not blinded to treatment condition, conducted the anthropometric measurements. Weight was measured using a digital Tanita scale to the nearest 0.1 kg. Height was measured using a wall-mounted stadiometer to the nearest 0.1 cm. The weight and height of mothers was converted to BMI (kg/m^2^) and weight was used to calculate percent weight loss. The height, weight, age, and gender of children was used to calculate BMI z-score using the Centers for Disease Control and Prevention growth curves [35].

#### Sugar-sweetened beverage intake/Diet

Mothers completed a single 24-h dietary recall for her own intake and another 24-h recall in which she reported her child’s intake at each assessment period. Trained research assistants that were blind to treatment group assignment completed the dietary recalls in order to avoid any biases. Because the goal of the Smart Moms study was to impact maternal practices of providing children with SSBs or juice within the home environment, the mother-reported child recall included only food and beverages that the parents provided the child. Any food or beverages purchased at or provided by childcare, school, or another care provider were not included in the dietary recall. The dietary data were entered into the Nutrition Data System for Research (NDSR), which was used to generate reports of the child’s SSB/juice intake in fluid ounces per day, mothers’ caloric beverage intake in ounces and calories per day, in addition to other dietary variables such as total calories, fat, and added sugars.

#### Physical activity

To assess maternal physical activity, mothers completed the Paffenbarger Physical Activity Questionnaire [36]. It has been used to assess leisure time activity in many weight loss trials and can be scored to provide an estimate of minutes per week and calories expended per week in overall leisure time activity. This questionnaire was administered during the in-person assessment visit by trained study staff. Child physical activity was assessed with the physical activity module of the Children’s Physical Activity Questionnaire [37]. This questionnaire was completed online and asked the mother to report, in the last 7 days, how many times and for how long the child did each of 28 activities. It has been validated against accelerometry in children ages 4–5, with a correlation of 0.42 between self-report measurement of MVPA and measurement of MVPA from accelerometers.

#### Social cognitive mediators

There are no existing validated questionnaires that measure theoretical constructs specific to parents reducing the SSB consumption of young children. Therefore, this study used a series of questions drawn from several previous research studies and developed specifically for use in this study. To assess self-efficacy for reducing their child’s SSB consumption and other behaviors, mothers completed six items from a previous study that assessed their confidence in making obesity-related changes with their children [38]. In the cited study, the items had high internal consistency (α = 0.72). An additional three items adapted from a previous study [39] were added to assess self-efficacy for making changes in the child’s SSBs and outcome expectations for limiting SSBs. Mothers’ frequency of setting limits with SSB consumption and other behaviors was assessed with nine items used and adapted from a previous study [40]. The questions assessed mothers’ agreement with restricting SSB/juice consumption, TV time, and fast food consumption.

#### Home environment

Mothers completed several online questionnaires that assessed the social and physical aspects of the food and physical activity home environments. The physical food environment was assessed using a measure developed for a previous family-based study [41], in which mothers reported whether or not each of 71 food items was present in the home in the last week. The social food environment was assessed with the Meals in Our Household questionnaire [42]. The questionnaire consisted of 50 items across 6 subscales: structure of family meals, problematic child mealtime behaviors, use of food as a reward, parental concern about child’s diet, spousal stress related to child’s mealtime behaviors, and influence of child’s food preferences on what other family members eat.

#### Maternal sedentary behavior

In addition, mothers’ self-report of sedentary behavior was measured with the Sedentary Behavior Questionnaire [43], which asks participants to report the number of hours they spend in a wide range of sedentary activities (watching TV, playing computer or video games, sitting down while listening to music, on the telephone, or doing work or crafts, and sitting down during transportation) on an average weekday and weekend day. In the validation sample, test-retest reliability coefficients ranged from 0.51 to 0.90.

#### Child screen time

Child screen time was measured with six screen time questions adapted from a well-known screen time intervention [44]. Mothers reported the child’s average total screen time over the prior 7 days (during the time that the child was home) for (1) TV/DVDs, (2) playing video games, (3) using the computer, and (4) viewing or playing games on a smartphone or tablet. Mothers estimated for each category the average hours per weekend day and average hours per weekday. The categories are summed together, and from that, an average amount of screen time per day will be calculated (Average screen time = [[weekday time × 5] + [weekend time × 2]]/7).

#### Parent feeding styles

The Parental Feeding Style Questionnaire [45] is a 27-item questionnaire that is used to assess four aspects of feeding style: emotional feeding, instrumental feeding, prompting/encouragement to eat, and control over eating.

#### Depression

Mothers’ depressive symptoms were assessed with the Center for Epidemiologic Studies Depression Scale Short Form, a 10-item self-report measure that measures depressive feelings and behaviors during the past 7 days [46].

### Data analysis

SAS version 9.3 (Cary, NC) will be used to conduct statistical analyses for the primary and secondary analyses of this intervention. Intent-to-treat analyses using linear mixed models will be used to examine changes in the primary and secondary outcomes. The linear mixed models will include a random intercept and effects for treatment group, time, and group by time interactions for both follow-up visits. A Bonferroni adjustment will be used to account for the effect of comparisons at multiple time points. Any characteristics found to be associated with both treatment group and the outcomes will be defined as confounders and will be included as covariates in analyses.

## Discussion

This study was designed to determine whether a low-burden intervention delivered using mobile methods and targeted towards mothers could be effective at changing child sugar-sweetened beverage intake. There is a critical gap in the literature such that few interventions have been successful at modifying parent and child weight-related behaviors. This is the first randomized controlled trial intended to determine the efficacy of an innovative mobile-based program to change behavior in mother-child dyads.

Though the study is innovative in its design and goals, there are several limitations. First, in order to limit measurement burden on mothers, the outcome of SSB/juice intake was measured by a 1-day 24-h dietary recall, versus the gold standard of 3days of dietary recall data. This is a limitation, as SSB/juice intake likely varies considerably from day to day. However, because the dietary recall data was being used to compare average dietary values between groups, intra-individual variability is less of an issue. Thus, the use of only one dietary recall in this study was the best choice for dietary and SSB/juice assessment in this population. Additionally, the self-reported nature of the SSB/juice and dietary data could be a potential limitation of the study, although use of the 24-h recall is the current standard in behavioral interventions, and research assistants were blinded to treatment group in order to mitigate the risk of bias.

Lastly, this intervention targeted solely an overweight or obese mother as the agent of change due to the well-known strength of association between child and maternal weight and dietary behaviors. However, the intervention did not directly target or involve any other family members living in the home, including fathers, partners, and other children. If the father is overweight or obese [47], and if there are additional children in the home who potentially increase parental barriers to making dietary and physical activity changes, then it may not be sufficient to target only the mother while overlooking the other family members.

Nonetheless, the innovative nature of the Smart Moms intervention will be informative for the literature. Given the rising proportion of preschoolers who are obese, and given that overweight and obese parents could benefit from programs targeted to the demands of having a family, there is a significant need for sustainable interventions that are effective and maximize adherence among parents. Smart Moms adapts the traditional behavioral weight loss intervention to focus on specific dietary behaviors, uses simplified self-monitoring, and delivers intervention materials via technology to reduce time demands and increase parental adherence. Thus, it will contribute to the emerging evidence base seeking to inform successful strategies to prevent childhood obesity through family-based approaches.

## Abbreviations

BMI: Body mass index
NDSR: Nutrition data system for research
RCT: Randomized controlled trial
SCT: Social cognitive theory
SSBs: Sugar-sweetened beverages.
